# Ion channels, long QT syndrome and arrhythmogenesis in ageing

**DOI:** 10.1111/1440-1681.12721

**Published:** 2017-09-20

**Authors:** Kamalan Jeevaratnam, Karan R Chadda, Samantha C Salvage, Haseeb Valli, Shiraz Ahmad, Andrew A Grace, Christopher L‐H Huang

**Affiliations:** ^1^ Faculty of Health and Medical Sciences University of Surrey Guildford UK; ^2^ School of Medicine Perdana University–Royal College of Surgeons Ireland Serdang Selangor Darul Ehsan Malaysia; ^3^ Physiological Laboratory University of Cambridge Cambridge UK; ^4^ Department of Biochemistry University of Cambridge Cambridge UK; ^5^ Division of Cardiovascular Biology Department of Biochemistry University of Cambridge Cambridge UK

**Keywords:** ageing, cardiac arrhythmia, fibrotic change, long QT syndrome, murine models, sodium channel

## Abstract

Ageing is associated with increased prevalences of both atrial and ventricular arrhythmias, reflecting disruption of the normal sequence of ion channel activation and inactivation generating the propagated cardiac action potential. Experimental models with specific ion channel genetic modifications have helped clarify the interacting functional roles of ion channels and how their dysregulation contributes to arrhythmogenic processes at the cellular and systems level. They have also investigated interactions between these ion channel abnormalities and age‐related processes in producing arrhythmic tendency. Previous reviews have explored the relationships between age and loss‐of‐function Na_v_1.5 mutations in producing arrhythmogenicity. The present review now explores complementary relationships arising from gain‐of‐function Na_v_1.5 mutations associated with long QT3 (LQTS3). LQTS3 patients show increased risks of life‐threatening ventricular arrhythmias, particularly after 40 years of age, consistent with such interactions between the ion channel abnormailities and ageing. In turn clinical evidence suggests that ageing is accompanied by structural, particularly fibrotic, as well as electrophysiological change. These abnormalities may result from biochemical changes producing low‐grade inflammation resulting from increased production of reactive oxygen species and superoxide. Experimental studies offer further insights into the underlying mechanisms underlying these phenotypes. Thus, studies in genetically modified murine models for LQTS implicated action potential recovery processes in arrhythmogenesis resulting from functional ion channel abnormalities. In addition, ageing wild type (WT) murine models demonstrated both ion channel alterations and fibrotic changes with ageing. Murine models then suggested evidence for interactions between ageing and ion channel mutations and provided insights into potential arrhythmic mechanisms inviting future exploration.

## 1 AGEING AND THE INCIDENCE OF CARDIAC ARRHYTHMIAS

Ageing refers to the normally occurring, physiological degeneration that causes a persistent and progressive decline in the fitness of an organism with time.^1^ This changing biological background increases the prevalence of a wide range of clinical conditions with age. Of these, cardiovascular disease is the leading cause of death in the elderly.^2^ At least some of this mortality has been attributed to the progressive deterioration in cellular and tissue function in the heart.^3^, ^4^ Among other effects, this increases the incidences of cardiac arrhythmias.^5^ Atrial fibrillation (AF) is the most common form of arrhythmia and results in substantial mortality and morbidity.^6^ Its adult prevalence rises from an overall level of around 1%‐4% to >13% in those over the age of 80 years.^7^ Similarly, the incidence of ventricular arrhythmias potentially resulting in sudden cardiac death also increases with age, showing higher male than female prevalence that converge by the eighth decade of life.^8^

Arrhythmic phenomena fundamentally arise from a disruption of the complexly interacting sequence of ion current activation and inactivation underlying action potential generation and propagation through successive regions of the heart. This involves a wide range of specific ion channel types and the effects upon them of their associated subunits. These channels variously mediate surface membrane sodium (Na^+^), potassium (K^+^), and calcium (Ca^2+^) currents, as well as Ca^2+^ fluxes between different intracellular compartments. Much detailed work has characterised their biophysical function in individual channels. However, less is known about the effects of abnormalities in the expression or properties of particular ion channels at the systems level. Nevertheless, alterations in particular genes encoding specific ion channels result in well‐defined arrhythmic conditions, and thereby provide useful clinical insights into how they might produce arrhythmic tendency. These in turn have prompted experimental studies then exploring for the underlying mechanisms. Of these, the Brugada (BrS) and long QT syndromes (LQTS) affect 35 in 100 000^9^ and 1 in 2500^10^ persons respectively. BrS is associated with Na_v_1.5 haploinsufficiency compromising Na^+^ channel activation and the resulting action potential propagation. Previous reviews have explored the relationships between ageing, underlying ion channel abnormalities, morphological changes and arrhythmic phenotypes in this condition.^11^ The present review goes on to summarise corresponding insights in LQTS, which exemplifies abnormal recovery from excitation.

## 2 ION CHANNEL INVOLVEMENT IN ARRHYTHMIA IN LQTS

Long QT syndrome is characterised by prolonged electrocardiographic (ECG) QT intervals reflecting increased ventricular action potential durations (APD) and, in some cases, aberrant T‐wave ECG signatures. This is associated with increased risks of ventricular arrhythmogenesis taking the form of episodic polymorphic ventricular tachycardias (VT) or torsades de pointes (TdP). These present either as self‐terminating syncopal episodes or may further degenerate into ventricular fibrillation and/or sudden cardiac death,^12^ which forms the first presenting event in approximately 5% of patients.^10^ Clinical studies additionally associated LQTS with increased risks of polymorphic atrial tachyarrhythmias. Such tachyarrhythmic episodes are observably longer in LQTS patients than other patients with persistent or induced AF, and where P wave features take on undulating characteristics. It is thought that the latter periodic changes in P wave vector during polymorphic atrial tachyarrhythmias resemble the twisting of the QRS axis observed in ventricular TdP, indicating an atrial form of TdP.^13^ The range of congenital LQT1‐LQT13 subtypes are each associated with particular mutations that can involve more than 15 genes.^12^ Where these involve loss‐of‐function mutations they usually concern K^+^ current expression or function, as in LQT1, LQT2, LQT5, LQT6, LQT7, LQT11 and LQT12.^14^ In contrast, gain‐of‐function mutations associated with LQTS often involve Na^+^ and Ca^2+^ current function, as in LQT3, LQT8, LQT9 and LQT10.^14^ LQT1, LQT2 and LQT3 are the most common LQTS variants and account for about 97% of the cases of congenital LQTS.^15^ These conditions have additional associations with other arrhythmic syndromes; thus sinoatrial node (SAN) dysfunction occurs in patients with both loss‐ and gain‐of‐function SCN5A mutations.^16^, ^17^

## 3 PRO‐ARRHYTHMIC CHANGES ASSOCIATED WITH AGEING

Ageing is itself accompanied by structural and biochemical changes that may themselves increase arrhythmic risk. The increased production of reactive oxygen species (ROS) and superoxide in cardiac tissue associated with oxidative stress and low‐grade inflammation promotes fibrotic change.^18^, ^19^, ^20^ In contrast, Na^+^ channel expression is conserved with age.^21^ Nevertheless age‐related fibrotic changes could disrupt connexin‐mediated cell‐cell coupling or result in fibroblast‐cardiomyocyte fusion. These would compromise AP conduction,^22^ increase the dispersion of repolarisation,^23^ and possibly prolong ventricular recovery times.^24^, ^25^, ^26^ All these factors can contribute arrhythmic substrate. Thus, normal cardiac ageing is accompanied by electrocardiographic increases in both QT dispersion (QTd), reflecting heterogeneities between maximum and minimum QT intervals^24^, ^27^ and QT interval, reflecting the time interval between myocardial depolarisation and repolarisation.^24^, ^28^ Arrhythmic risk increases by about 5% for every 10‐ms increase in QT interval beyond the upper normal limit^29^ and it is also affected by heterogeneity of repolarisation.

## 4 CLINICAL INSIGHTS ON THE INTERACTIONS BETWEEN AGEING, GENDER AND LQTS PHENOTYPES

Several LQTS phenotypes vary with age, potentially providing platforms for exploring the effect of age on ion channel properties and their implications for arrhythmic tendency. In addition, the genetic abnormalities and age might interact and thereby accentuate any morphological phenotypes,^30^, ^31^ as occurs for the fibrotic phenotype associated with channelopathies such as BrS.^32^ LQTS3 patients are frequently bradycardic, and this adds to the increased atrial and ventricular arrhythmic risks known to be associated with the lower heart rates occurring during rest and sleep. LQTS patients show lower ages of onset for AF, typically at age 50±14 years.^33^, ^34^ LQTS, particularly LQTS3, patients show increased risks of life‐threatening ventricular arrhythmias after 40 years of age that are influenced by particular specific factors, such as gender and clinical history.^12^, ^35^ LQTS3 patients can also show long‐term changes normally associated with Na_v_1.5 haplo‐insufficiency resulting in overlap syndromes that combine both loss (BrS) and Na_v_1.5 gain‐of‐function (LQTS3) phenotypes. For example, an eight‐generation family carrying *SCN5A*+/1795insD showed bradycardia and TdP episodes, characteristic of LQTS3, and ECG ST segment elevation and high rates of nocturnal SCD, characteristic of BrS.^36^ In addition to the effects of ageing, gender can affect LQTS phenotypes: women show longer QTc intervals than men, increasing predisposition to polymorphic ventricular tachycardia.^37^, ^38^ In LQTS1 and LQTS2, women are at higher risk of TdP than men. The gender difference does not appear to apply to LQTS3 induced arrhythmia.^39^ Nevertheless, whilst their prolonged QTc is linked to increased arrhythmic incidences, women show a lower likelihood of sudden cardiac death than men, especially through their reproductive years. Given these gender‐related differences in QTc duration, the diagnosis of LQTS is sex‐specific with QTc durations of >460 ms in females and >440 ms in males.^40^

## 5 EXPERIMENTAL STUDIES OF ARRHYTHMIC PHENOTYPES

Experimental studies of arrhythmogenic mechanisms for LQTS and their relationship to ageing have employed a range of animal systems each with their own limitations, particularly bearing upon the availability or otherwise of genetically modified variants modelling the corresponding human conditions. Primate studies, such as those on female Cynomolgus monkeys, demonstrated associations between age and QT prolongation, whether in the presence or absence of pharmacological intervention.^41^ Equine wedge preparations confirmed central roles for K_v_11.1 in repolarisation processes in common with human hearts.^42^ Ageing increased QT intervals and susceptibility to E‐4031 or terfenadine‐induced QT prolongation in conscious female guinea pigs,^43^ though not to cisapride‐induced QT prolongation in isolated guinea pig hearts.^44^ Transgenic LQT2 and LQT3 rabbit models have helped clarify roles of spatial and temporal dispersions of repolarisation as arrhythmogenic substrates^45^ and the effects of potential anti‐arrhythmic agents, such as free polyunsaturated fatty acids, in preventing TdP.^46^

Mice currently provide the main transgenic system for studying cardiac arrhythmias. They reproduce at short intervals with relatively brief (~20 day) gestations, facilitating provision of aged mice over relatively short time periods.^11^ Mouse and human hearts have anatomically similar conducting, sinoatrial and atrioventricular nodes, His‐Purkinje systems and contracting atrial and ventricular chambers. Similarities in their physiological properties include their Na^**+**^ channel characteristics and their Na_v_1.5‐mediated phase 0 depolarisation phases initiating electrophysiological activity. They differ in their >10 times faster resting heart rates, smaller L‐type Ca^2+^ currents, and differing K^+^ channel contributions to action potential recovery.^47^, ^48^, ^49^, ^50^ Nevertheless, the murine model has been useful for investigation of arrhythmic conditions related to abnormalities in Na^+^ channel characteristics.^51^, ^52^

## 6 THE MURINE *SCN5A+/∆KPQ* SYSTEM AS A MODEL FOR LQTS3

The murine *Scn5a+/∆KPQ* heart has been used to explore arrhythmic mechanisms^60^ and the effects upon these of pharmacological interventions, in LQTS3.^53^ *Scn5a+/∆KPQ* hearts carry a gain‐of‐function mutation deleting three conserved amino acids (Lys‐1505, Pro‐1506, Gln‐1507) within the *Scn5a* inactivation domain, disrupting Na_v_1.5 inactivation kinetics.^54^ This enhances late sodium currents (*I*_NaL_), elongating the AP plateau and increasing the likelihood of early afterdepolarisation (EAD) phenomena that can precipitate TdP episodes.^55^, ^56^ Isolated, Langendorff‐perfused, *Scn5a+/∆KPQ* ventricles recapitulated increased ventricular arrhythmic tendencies and electrophysiological features established in clinical LQTS3. They showed prolonged repolarisation time courses preferentially affecting epicardial as opposed to endocardial APD. This inverted the transmural repolarisation gradients normally observed in wild type (WT) hearts.^57^ There was also a potential mismatch between AP recovery to the resting membrane potential and of the recovery of excitability as reflected in the effective refractory periods (ERP), as quantified by APD/ERP ratios. This produced a substrate in which triggering by extrasystolic stimulation elicited sustained arrhythmia: both extrasystolic stimuli at progressively decreased intervals following regular pacing trains and abrupt increases in pacing rate increased arrhythmic incidences.^56^, ^58^, ^59^, ^60^ Atrial *Scn5a+/∆KPQ* cardiomyocytes similarly showed prolonged APD and frequent EADs rescued by the *I*_NaL_ inhibitor ranolazine,^61^, ^62^ particularly at slow pacing rates.^54^, ^63^

Murine *Scn5a+/∆KPQ* hearts also recapitulated the clinical pharmacological features of clinical LQTS3. Flecainide and quinidine respectively exerted anti‐ and pro‐arrhythmic effects in *Scn5a+/∆KPQ* ventricles. The observed arrhythmogenicity with quinidine challenge correlated with accentuated *I*_NaL_ and EAD phenomena that could potentially trigger spontaneous arrhythmia. Arrhythmic tendency in murine *Scn5a+/∆KPQ* ventricles could then be accounted for by triggering events following the appearance of EADs involving contributions from altered Ca^2+^ homeostasis, and from substrate sustaining the arrhythmia following such triggering. Thus, the dihydropyridine L‐type Ca^2+^ channel antagonist nifedipine (10 nmol/L‐1 μmol/L) decreased the incidences of both EADs and arrhythmias without altering APD through inhibiting *I*_CaL_ but not *I*_Na_.^57^, ^64^ The β‐adrenoceptor antagonist, propranolol, suppressed EADs and reduced epicardial APD whilst suppressing both spontaneous and provoked arrhythmias at 100 nmol/L concentrations.^65^ However, whilst 1 mmol/L concentrations also eliminated both EADs and spontaneous arrhythmias, it prolonged epicardial and reduced endocardial APDs. It also increased the incidences of arrhythmia following extrasystolic stimulation.^65^ Clinical studies similarly report that β‐adrenoceptor antagonism is less effective in suppressing arrhythmia in LQTS3 than in LQTS1 and LQTS2.^66^

Finally, resting membrane potential stabilisation by the K_ATP_ channel opener nicorandil^67^ reduced arrhythmic frequencies provoked by extrasystolic stimuli whilst reducing left ventricular (LV) epicardial but not LV endocardial APD in *Scn5a*+*/ΔKPQ* ventricles. It restored the transmural repolarisation gradients to those of normal (WT) hearts.^68^ Nicorandil is similarly anti‐arrhythmic in clinical LQTS, reducing QT intervals and spatial repolarisation gradients.^69^, ^70^, ^71^ Restitution properties investigated through progressive increases in pacing frequency of murine *Scn5a+/∆KPQ* hearts showed higher diastolic intervals following action potential recovery, DI_crit_, at which instabilities in excitation could potentially result in APD alternans producing re‐entrant substrate, compared to WT. These were further increased by quinidine and decreased by flecainide and nicorandil in parallel with their pro‐and anti‐arrhythmic effects.^68^, ^72^

## 7 MURINE MODELS FOR AGE‐DEPENDENT ARRHYTHMOGENICITY

Murine hearts similarly model cardiac changes with ageing. Firstly, ageing appears to be intrinsically associated with electrophysiological change. Aged mice (≥24 months) demonstrated 2.6‐fold higher frequencies of arrhythmic events.^21^ Surface ECG studies in both anaesthetised and ambulant mice and in isolated perfused hearts demonstrated increased PR and QT intervals at ≥25 months, reflecting prolonged atrioventricular conduction and ventricular repolarisation respectively. Isolated hearts showed prolonged ventricular mean APDs. These findings could be explained in terms of reduced expression of voltage‐gated K^+^ currents (*I*_to_, *I*_K,slow1_, *I*_K,slow2_ and *I*_ss_) in LV myocytes despite an increased *I*_NaL_ from old 31‐32 month mice. These findings in turn correlated with reduced K_v_1.4 and K_v_1.5 but normal Na_v_1.5 expression.^21^ Secondly, aged mice (52 weeks) showed progressive myocardial fibrosis, which was reduced by inhibiting the renin‐angiotensin‐aldosterone system. Chronic administration of eplerenone and losartan, whether alone or in combination, reduced both interstitial fibrosis in the RV and LV and the occurrence of scattered patches of replacement fibrosis, as revealed by Sirius staining for collagen. Ventricular epicardial mapping in Langendorff‐perfused hearts demonstrated a correspondingly reduced arrhythmic inducibility to extrasystolic stimulation and burst pacing that correlated particularly with reductions in the patchy fibrosis. This was accompanied by increased RV transverse conduction velocities and decreased anisotropic ratio between the transverse and longitudinal velocities.^73^

Atria of aged (24 month) male Kunming mice showed a greater inducibility of AF, and longer electrocardiographic P‐wave duration and sinus node recovery times, than their younger (2 month) counterparts. There were accompanying increased dispersions of repolarisation and greater *I*_to_, though unchanged *I*_Kur_, particularly in the left atrium. Collagen estimations suggested an increased fibrotic phenotype,^74^ which might itself exert pro‐arrhythmic actions. Inactivation of murine atrial cardiomyocyte Mkk4 (*Mkk4*‐ACKO) increased interstitial fibrosis and transforming growth factor‐β1 (TGF‐β1) signalling with a dysregulation of matrix metalloproteinases, particularly in ageing (1 year) mice compared to adult (6 month) and young (3‐4 month) littermates. It increased the sensitivity of cultured cardiomyocytes to angiotensin II‐induced activation of TGF‐β1 signalling. The aged *Mkk4*‐ACKO mice were more susceptible to atrial tachyarrhythmias than the corresponding *Mkk4*‐F/F mice. This correlated with observations of slowed and dispersed atrial conduction which modelling studies related to arrhythmic effects. Human atrial tissues in AF similarly showed Mkk4 downregulation together with increased production of profibrotic molecules compared to results from subjects in sinus rhythm.^75^

## 8 EXPERIMENTAL INSIGHTS ON THE INTERACTIONS BETWEEN AGEING AND LQTS PHENOTYPES

Recent reports suggest that murine hearts may also model interactions between age‐related electrophysiological and morphological changes and particular genetic alterations in specific ion channels related to LQTS. Thus, extrasystolic stimulation experiments demonstrated that young (3 month) and adult (5‐9 months) *Scn5a*+/∆KPQ hearts showed no increases in atrial arrhythmic tendency compared to WT.^54^, ^76^ Indeed, with pacing at high stimulus voltages, <9 month *Scn5a+/∆KPQ* hearts showed lower incidences of atrial arrhythmic episodes, which had shorter durations, than WT following extrasystolic stimulation and burst pacing.^54^ In contrast, arrhythmic tendencies in aged (12 month) *Scn5a+/∆KPQ* mice were greater than in either young or aged WT mice.^76^ These findings correlated with the following differences between experimental groups: (i) Regularly paced *Scn5a+/∆KPQ* hearts showed longer atrial APDs and P wave durations than WT hearts, and this difference increased with age.^54^, ^76^ (ii) Young WT and young *Scn5a+/∆KPQ* showed similar AERPs, whereas aged WT but not aged *Scn5a+/∆KPQ* showed increased AERPs. (iii) In consequence, aged *Scn5a+/∆KPQ* showed the greatest APD/AERP ratios potentially resulting in arrhythmic substrate. (iv) These findings were consistent with the greater Na_v_1.5 expression in young *Scn5a+/∆KPQ* than young WT. (v) Na_v_1.5 expression then increased with age in the WT but not the *Scn5a+/∆KPQ*.^76^

*Scn5a+/ΔKPQ* mice also showed compromised pacemaker activity, reflected in frequent episodes of sinus bradycardia, sinus pause/arrest, and longer sinus node recovery times. These phenotypic characteristics resemble those seen in sick sinus syndrome (SSS), which can occur at any age but is commonly associated with the elderly.^77^ Additionally, these findings were associated with electrocardiographic evidence for depressed intra‐atrial, atrioventricular node, and intraventricular conduction. These findings were corroborated in isolated *Scn5a+/ΔKPQ* sinoatrial preparations which, compared to wild‐type preparations, showed reduced intrinsic heart rates and slower conduction from the SAN to surrounding atrium. Computer simulations of single SAN cells and two‐dimensional SAN‐atrial models attributed these findings to a combination of augmented *I*_NaL_ and reduced total *I*_Na_.^78^

Comparable changes resulting in a similar overlap syndrome occur in murine *Scn5a+/1795insD* hearts which combine increased QTc intervals with slowed ventricular conduction similarly attributable to reduced *I*_Na_. ECG studies revealed reduced sinus rates, bradycardic pauses that could exceed 500 ms and increased PQ intervals and QRS durations. Patch‐clamped ventricular *Scn5a+/1795insD* myocytes showed action potential prolongation and increased *I*_NaL_ despite normal voltage‐dependent Na_v_1.5 activation, steady‐state rapid or slow inactivation properties and recovery from inactivation, with the expected action potential prolongation. However, they also exhibited evidence for Na_v_1.5 deficiency in the form of marked (~40%) reductions in peak *I*_Na_ and rate of rise of their action potentials (d*V*/d*t*)_max._ Epicardial multi‐electrode array recordings in Langendorff‐perfused *Scn5a+/1795insD* hearts confirmed a slowed conduction of excitation.^79^

Finally, recent studies associated a development of fibrotic change with LQTS3. The characteristics of murine F1759A‐dTG atria aged between 4‐12 weeks were consistent with an altered genotype affecting the fibrotic process itself. The mutation was associated with clinical AF. The murine hearts showed an incomplete Na_v_1.5 inactivation increasing *I*_NaL_ and resulting in a prolonged APD and prolonged episodes of spontaneous AF that demonstrated atrial rotors, waves and wavelets resembling AF. There was an accompanying fibrosis, myofibril disarray, mitochondrial dysfunction and atrial and ventricular enlargement.^80^ The relationship between pacemaker dysfunction and the observed phenotypic characteristics has been largely attributed to *I*_NaL_ and *I*_Na_ with no reference made to the funny current (*I*_f_). It is possible that with ageing the observed phenotypic changes may additionally be attributable to *I*_f_ in LQTS3. A study exploring the effects of ageing on *I*_f_ suggest that there is reduced atrial mRNA and protein expression of the hyperpolarisation‐activated cyclic nucleotide‐gated channel (HCN) isoforms HCN2 and HCN4 in aged dogs.^81^

## 9 CONCLUSIONS

**Figure 1:**
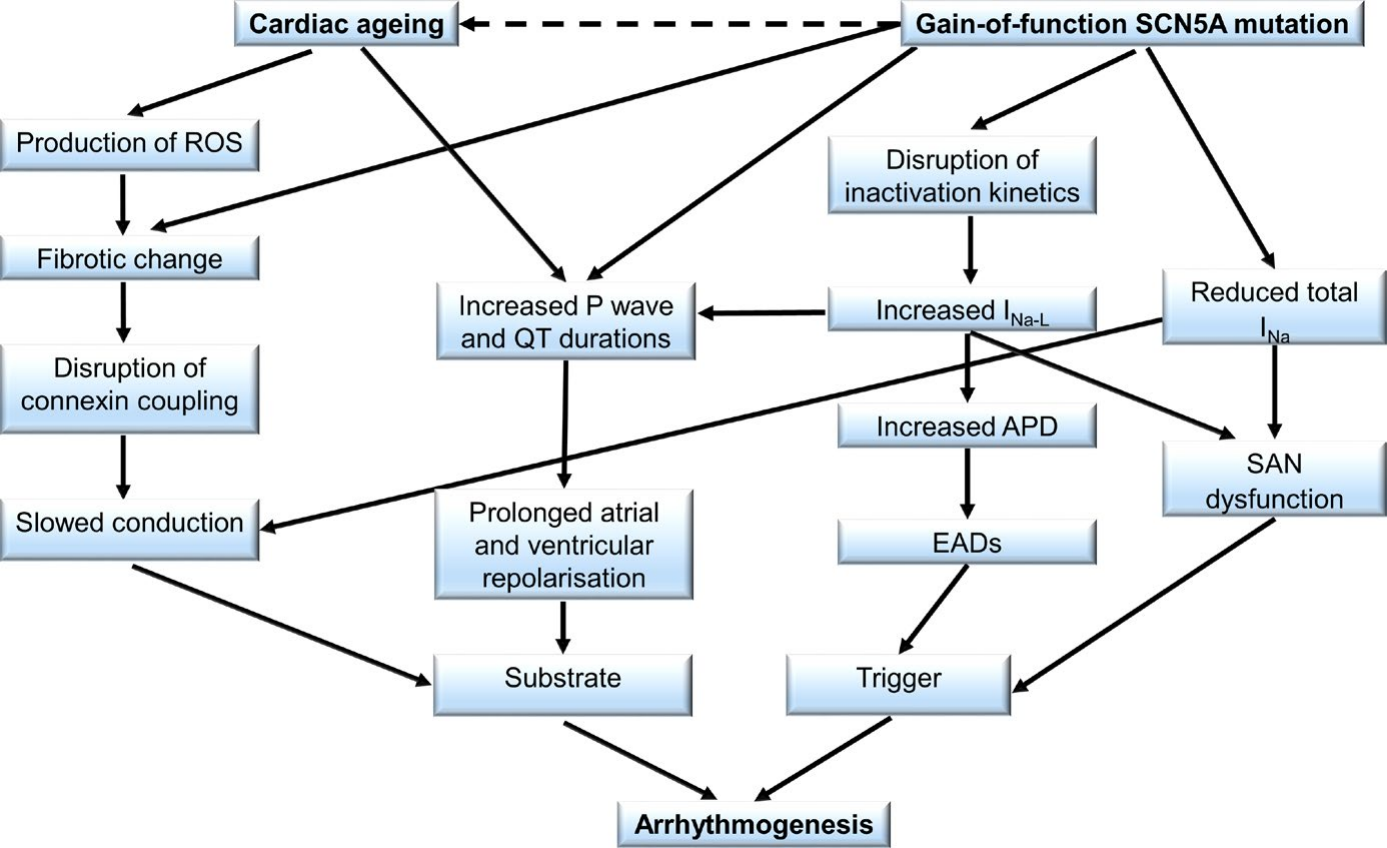
Unified diagram summarising the superimposition of a cardiac ageing phenomenon on a gain‐of‐function SCN5A mutation. An increased production of reactive oxygen species is observed with cardiac ageing, causing low‐grade inflammation that promotes fibrotic change. Experimentally, a gain‐of‐function Scn5a mutation has also been associated with fibrotic change. Through this fibrotic change, cardiac ageing and a gain‐of‐function SCN5A mutation converge on disrupting connexin coupling between myocytes. Cardiac ageing additionally leads to increased P wave and QT durations prolonging the repolarisation of both the atria and ventricles. Collectively these create substrates for arrhythmogenesis. The gain‐of‐function SCN5A mutation delays channel inactivation, resulting in an increased late sodium current, forming a substrate for arrhythmogenesis through prolongation of atrial and ventricular repolarisation or forming triggers through the promotion of early after depolarisations or sino‐atrial node dysfunction. The dotted arrow represents a hypothetical possibility that a gain‐of‐function mutation may accelerate cardiac ageing processes, leading to arrhythmic tendencies earlier in life

Arrhythmias result from disruptions in the orderly process of ion channel activation and inactivation underlying the action potential initiation and propagation through cardiac tissue. The ion channelopathies related to LQTS have provided useful illustrative examples that have facilitated our understanding of the roles that ion channels abnormalities have in arrhythmogenic processes. As summarised in Figure 1, we review the physiological background underlying the increased incidence of atrial and ventricular arrhythmias with age in a gain‐of‐function Na^+^ channel mutation attributable to LQTS3. This demonstrates how cardiac ageing and a gain‐of‐function mutation converge to exert differing and interacting mechanisms, which lead to both trigger and substrate components for arrhythmogenesis. Clinical studies have also clarified the background of structural, particularly fibrotic, as well as the biochemical and electrophysiological changes that occur with ageing. The mechanisms by which such changes exert pro‐arrhythmic effects have been clarified by experimental studies which suggest both alterations in recovery and activation properties of the heart. It was possible to enumerate physiological changes occurring in genetically modified murine models for LQTS, in particular LQTS3, and the physiological and fibrotic changes in ageing WT, as well as to explore examples where these changes might interact. This provides possible directions for exploring the relationship between age‐related changes and arrhythmia. Furthermore, though not exclusive to cardiac channelopathies, an increasing ageing population necessitates explorations of the relationship between age‐related changes and choices of clinical therapeutic interventions. Presently there is a paucity of scientific evidence as to how ageing influences the effectiveness of therapeutic interventions and their related complications, specifically in LQTS3. The present review provides evidence of how cardiac ageing leads to structural and electrophysiological remodelling of ion channels. Therefore, the age‐related remodelling changes could well alter the effects of anti‐arrhythmic agents targeting ion channels. This may result in age‐specific indications for different available therapeutic interventions directed at cardiac electrophysiological abnormalities. Finally, targeting mechanistic pathways leading to fibrosis and ROS generation associated with the ageing process itself, could also contribute to reducing arrhythmic tendency.

## DISCLOSURES

None declared.
